# Mining the Biomarker Potential of the Urine Peptidome: From Amino Acids Properties to Proteases

**DOI:** 10.3390/ijms22115940

**Published:** 2021-05-31

**Authors:** Fábio Trindade, António S. Barros, Jéssica Silva, Antonia Vlahou, Inês Falcão-Pires, Sofia Guedes, Carla Vitorino, Rita Ferreira, Adelino Leite-Moreira, Francisco Amado, Rui Vitorino

**Affiliations:** 1 UnIC—Cardiovascular Research and Development Centre, Department of Surgery and Physiology, Faculty of Medicine, University of Porto, 4200-319 Porto, Portugal; asbarros@med.up.pt (A.S.B.); ipires@med.up.pt (I.F.-P.); amoreira@med.up.pt (A.L.-M.); 2 iBiMED—Department of Medical Sciences, Institute of Biomedicine, University of Aveiro, 3810-193 Aveiro, Portugal; jessica.ferreira.da.silva.18@gmail.com; 3 Biotechnology Division, Biomedical Research Foundation of the Academy of Athens, 115 27 Athens, Greece; vlahoua@bioacademy.gr; 4 LAQV-REQUIMTE, Departamento de Química, Universidade de Aveiro, 3810-193 Aveiro, Portugal; sguedes@ua.pt (S.G.); ritaferreira@ua.pt (R.F.); famado@ua.pt (F.A.); 5 Faculty of Pharmacy, University of Coimbra, 3000-548 Coimbra, Portugal; csvitorino@ff.uc.pt; 6 Coimbra Chemistry Centre, Department of Chemistry, University of Coimbra, 3004-535 Coimbra, Portugal; 7 Center for Neurosciences and Cell Biology (CNC), University of Coimbra, 3004-504 Coimbra, Portugal

**Keywords:** urine, peptides, proteases, peptidomics, degradomics, biomarkers, predictive, preventive and personalized (3P) medicine, molecular patterns, individualized patient profiling

## Abstract

Native biofluid peptides offer important information about diseases, holding promise as biomarkers. Particularly, the non-invasive nature of urine sampling, and its high peptide concentration, make urine peptidomics a useful strategy to study the pathogenesis of renal conditions. Moreover, the high number of detectable peptides as well as their specificity set the ground for the expansion of urine peptidomics to the identification of surrogate biomarkers for extra-renal diseases. Peptidomics further allows the prediction of proteases (degradomics), frequently dysregulated in disease, providing a complimentary source of information on disease pathogenesis and biomarkers. Then, what does urine peptidomics tell us so far? In this paper, we appraise the value of urine peptidomics in biomarker research through a comprehensive analysis of all datasets available to date. We have mined > 50 papers, addressing > 30 different conditions, comprising > 4700 unique peptides. Bioinformatic tools were used to reanalyze peptide profiles aiming at identifying disease fingerprints, to uncover hidden disease-specific peptides physicochemical properties and to predict the most active proteases associated with their generation. The molecular patterns found in this study may be further validated in the future as disease biomarker not only for kidney diseases but also for extra-renal conditions, as a step forward towards the implementation of a paradigm of predictive, preventive and personalized (3P) medicine.

## 1. Introduction

The peptidome refers to the whole set of low molecular weight (LMW) peptide elements in a sample. The peptidome is often viewed as a subset of the proteome since most polypeptides (generally with a molecular weight lower than 20 kDa) result from the cleavage of native proteins [1,2,3]. One of the key features of peptidomics, i.e., the analysis of the native peptidome, is studying endogenous proteolytic events while preserving information on post-translational modifications [4]. Proteolytic cleavage is an essential biological process for protein maturation, activation, and turnover (lysosomal and proteasomal degradation) [5]. The human degradome comprises over 550 proteases, categorized into five groups, according to the catalytic mechanism [6]: serine proteases, threonine proteases, aspartic proteases, cysteine proteases, and metalloproteases. Many disease states are associated with an unrestricted and abnormal progression of proteolysis [7], making proteases and peptides key molecular entities with value as biomarkers and potential therapeutic targets. Peptidomics and degradomics hold, thus, great interest in predictive, preventive and personalized medicine (3P) medicine, the new paradigm in the practice of medicine, encompassing both “health care” and “disease care”, synergizing the advantages of several biomedical fields and cutting-edge technologies (such as mass spectrometry), through the collaboration of multidisciplinary teams [8].

There are many advantages of peptidomics over proteomics: (1) it can attain a larger number of disease-specific analytes, provided the generation of many peptides from a single parent protein, which results in improved discrimination; (2) it allows the study of the disease microenvironment, as peptides can cross endothelial barriers providing clinically-relevant biomarkers; (3) it is possible to extract information associated to proteolytic activity; (4) sample processing is much simpler, excusing enzymatic digestion and reducing intra-sample heterogeneity [9,10].

Endogenous peptides have only recently received attention as their relevance in disease characterization has not been adequately acknowledged [10]. Analytical techniques related to the characterization of the LMW sub-proteome lacked sensitivity at the dawn of proteomics [11]. However, today, the combination of high-performance liquid chromatography or capillary electrophoresis with high resolution mass spectrometers, usually using time-of-flight analyzers, but also Orbitraps, provides the needed sensitivity for the identification of thousands of peptides. The developments in the analytical platforms for the analysis of the peptidome is beyond the scope of this paper, but the reader is referred to two excellent reviews by Schrader [12] and Latosinska et al. [9]. Another major hindrance in peptidomics research is the low abundance of peptides in some, but not all, biological samples [7]. Furthermore, the detection of LMW protein species might be hampered by the presence of highly abundant proteins in some biofluids, such as blood, thus requiring pre-fractionation or enrichment steps [13]. Some endogenous peptides can also be shared over several proteins, obfuscating their role in disease etiology [14]. Besides, they require complex bioinformatics pipelines for analysis [10].

### The Potential of Urine Peptidomics

Urine is perhaps, of all biofluids, the one with greater potential in clinical peptidomics. Urinary proteins and peptides originate from the secretions of renal tubular epithelial cells, shedding of cells along the urinary tract, exosome secretion, and more importantly, from glomerular filtration of plasma [15,16]. Therefore, beyond the renal system’s pathophysiological status, the urinary peptidome is influenced by systemic disturbances. Due to the high reabsorption levels by the epithelial cells lining on the proximal tubules, protein species in the tubular lumen are present in minimal quantities [5]. Thus, peptides dominate a relatively larger proportion of the proteome in urine as opposed to other biofluids. Finally, the urine peptidome is considered more stable than other peptidomes, such as blood, because the bulk of proteolytic events has already been completed within the bladder [5].

The particular clinical value of urine peptidome is reflected by several studies where putative biomarker panels have been developed based on urinary peptides, aiming at the non-invasive diagnosis, prognosis of specific diseases, or flagging the need of an intervention. Due to anatomical proximity, most studies have centered, thus far, on the potential of urine peptidome to uncover biomarkers for kidney or urogenital conditions [17]. For instance, peptidomics has been used for the investigation of renal system diseases such as for the prediction of chronic kidney disease (CKD) progression or remission [16,18], suggesting associations of multiple peptides, predominantly collagen fragments, with glomerular filtration and the processes of inflammation and repair [19]. Another example is the so-called CKD273 classifier, a panel composed of 273 urinary peptides that has been validated in multiple studies for early detection of CKD and monitoring progression [18,20] and even recently used in a clinical trial for diabetic patient stratification and early detection of diabetic nephropathy [21]. Markoska et al. (2016) also studied peptidome alterations related to Autosomal dominant polycystic kidney disease (ADPKD) and acute kidney injury (AKI) and proposed a biomarker panel of 20 LMW urine peptides for ADPKD and 39 for AKI [22]. These approaches have high clinical relevance as CKD progression does not present early warning signs of kidney damage [16], hence reliable tools for early detection are deemed necessary for timely and therapeutic intervention. The search for disease markers [14,23] resulted for instance, in a peptide classifier composed of 21 peptides for diabetic nephropathy (DN) diagnosis showing a higher association with in situ glomerular lesions when compared to albuminuria (a commonly used biomarker in the clinics) [14]. Several authors also investigated DN using urinary peptidomics to unravel the disease pathophysiology mechanisms [24,25,26,27,28,29]. Urine peptidome is also an exciting study area for renal and genital cancers [17]. Based on the established link between lower urinary tract symptoms (LUTS) and prostate cancer (PCa), researchers have sought biomarkers able to differentiate between benign and malignant PCa in patients with a diagnosis of LUTS [30]. Potential biomarkers for bladder cancer have been intensively probed through urine analysis [10]. Furthermore, peptide profiling was proposed for the accurate prognosis and diagnosis of renal cell carcinoma (RCC) subtypes: papillary, chromophobe, and clear cell RCC [31].

Apart from the proven value of urine peptidomics in developing new diagnostic tools for diseases afflicting the renal-urogenital axis, the diagnostic/prognostic potential of urine peptidomics extends to other systemic conditions. Endogenous peptides from other sources may cross the endothelial barrier and be cleared out by the renal system, thus being surveyed through urine analysis [16,32,33]. For example, a set of 47 urinary peptides (mostly collagen-related) was found relevant to systemic lupus erythematosus progression and are candidates for a biomarker diagnosis panel [34]. In turn, 112 urinary peptides (also collagen-related) and 32 proteases (matrix metalloproteases, MMPs, and cathepsins) were associated with the molecular mechanisms of aging [35]. Besides, a 96-peptide classifier—the heart failure predictor—was proposed to stratify the risk for left ventricular heart failure [36]. It should be highlighted that urine’s intrinsically stable proteolytic activity makes this biological matrix particularly suited to study proteolysis [32], a hallmark of several conditions, such as metastasizing cancer. On top of peptides, proteases predicted and validated from urine peptidome may improve our perception of the disease and contribute with new insight and tools for the diagnosis/prognosis.

Clinical interest in urine peptides is mounting, particularly as surrogate biomarkers for chronic conditions, such as those directly afflicting the kidney. With the growing number of studies following a urine peptidomics approach, it is mandatory to evaluate the data published thus far to typify the major findings in this field, aiming to accelerate the introduction of urine peptides as biomarkers in clinical practice. By doing an unbiased collection of all studies until today, mining the accessible peptidome data, and comparing the peptidome profile across different disease classes, we aimed to highlight the disease-associated peptide markers currently established. Furthermore, by exploring peptides’ physical properties, such as mass, length, or isoelectric point, we hope to disclose disease class patterns that may be of diagnosis relevance upon a simple urinalysis. Finally, considering the urine’s unique proteolytic environment, which deems the urine peptide collections more stable than in any other biofluid, we might predict potentially dysregulated proteases in the various conditions, hopefully pinpointing new biomarkers to be added to the set of urinary peptides.

## 2. Brief Methods

### 2.1. Literature Search and Data Mining

A literature data research was conducted using the PubMed database up to July 2020, employing the combination of the following keywords: (“peptidome” OR “peptidomic” OR “peptidomics”) AND (“urine” OR “urinary”). This search retrieved 132 articles. After excluding reviews, articles written in languages other than English and not directly related to the urinary peptidome, and after including some articles found by cross-referencing the identified papers, a final set of 54 was obtained (Table S1). These studies covered over 30 diseases, categorized in nine main classes, from the most to the least frequent: renal, cardiovascular, cancer, autoimmune, metabolic, infections, mental, bowel, and respiratory diseases. Not surprisingly, studies on urinary peptidome in renal diseases represent the largest fraction (~36%), closely followed by those on cardiovascular abnormalities (~20%).

Each study was mined to extract the pathology, disease class, general information about the discovery and validation phases (e.g., number of subjects, number of identified proteins and peptides, number of sequenced differentially expressed peptides, and number of sequenced signature peptides), methodological approach (identification strategy, sequencing strategy, and biomarker assessment algorithm) and, if present, the details on the peptide panel performance in terms of C-statistics. The extracted data is available in Table S1 and represents all studies performed so far on urinary peptidomics, to the best of our knowledge. Then, to probe the hypothetical diagnostic nature of the peptides across all conditions/disease classes, the peptides displaying a potential discrimination power amongst conditions were selected from the discovery phase or from the validation phase, for instance, in the cases where the former were not available. In any case, the pool of peptides selected for analysis is marked as green in the Table S1, and the selection is justified in Table S2. The peptide sequences, together with the start and end amino acid positions and the source proteins (UniProt code and gene name), can be found in Table S3. Herein, only successfully sequenced peptides, showing relevant differences between the control condition and the disease, and whose sequence information was reviewed, according to UniProt, were considered. For comparison to the healthy peptidome, we had available two studies. Siwy et al. [37] could identify a total of 953 peptides, from over 13,000 urine samples belonging to either ill or control subjects. However, controls included both healthy subjects and other conditions not directly related to the disease at scope, which hindered the extraction of the “healthy” peptidome. Therefore, we could not use this dataset. Instead, we sourced the data of Di Meo et al. [38], the most detailed data on the human natural urine peptidome released and available to date. Moreover, this dataset presented over 4500 sequenced peptides, reflecting perhaps the utilization of a more advanced mass spectrometer, the Q exactive^TM^ instrument, not implemented at the time of the former publication (2011). The sequence information and parent proteins can be consulted in Table S4. As before, the peptides were selected only if they were derived from a reviewed protein sequence, according to UniProt.

### 2.2. Statistical and Bioinformatics Analyses

All peptide sequences associated with a disease were given a unique peptide ID and were associated to a specific condition, classified in nine major classes: autoimmune, bowel, cancer, cardiovascular, infection, mental, metabolic, renal, and respiratory diseases (see Table S3). Similarly, all peptides identified in the Di Meo et al. [38] dataset were given a unique peptide ID and associated to a single class: health.

Aiming to identify peptide signatures for each condition, we performed network analysis using Cytoscape (v.3.8.2). Briefly, an interaction table was created (Table S5), listing all disease-peptide associations, which was then imported to the Cytoscape environment. The conditions were defined as source nodes and the sequences as target nodes. We then mapped the nodes according to the degree, i.e., the total number of interactions, to single out those peptides associated with a unique condition (degree = 1). The node size was defined in descending order of the degree (with a bypass for the conditions themselves) to highlight those peptides with higher potential to create a disease fingerprint. The network analysis table was then exported, and the signature peptides obtained by filtering those of degree 1.

The signature peptides obtained through network analysis were then studied regarding their single amino acid composition and physical-chemical properties, using R statistical software built-in functions and the package ‘Peptides’ [39]. For this analysis we only considered disease classes with at least 20 unique peptides associated. As a reference, we used the complete set of peptides identified in healthy individuals [38]. First, we performed contingency analysis of the amino acid composition. Then, we assessed the percentage of nine amino acid groups, showing different physical-chemical properties: acidic, aromatic, basic, charged, hydrophobic, polar and charged, polar non-charged, small, and tiny amino acids. Finally, we calculated the sequence length (function “lengthpep”), molecular weight (function “mw”), isoelectric point (function “pI”), grand average of hydropathy (GRAVY) score (function “hydrophobicity”), in addition to the probability of antimicrobial activity (using the Collection of Antimicrobial Peptides (CAMP R3)’s prediction tool available on http://www.camp3.bicnirrh.res.in/, accessed on 23 July 2020) and the content in proline, an amino acid with special properties.

Provided the possibility to study the urine degradome, based on its peptidome, we used Proteasix web tool, an open-source peptide-centric tool, to compute the cleavage probability of native proteins originating the urinary peptides (Tables S3 and S4) by a wide array of proteases [40]. This algorithm was applied to both the disease (3014) and the health peptides (3767), aiming at predicting the most enriched proteases in all conditions and disease classes, whose urine peptidome has been characterized to date. In this analysis, both N-terminal and C-terminal cleavages were considered, but exclusively by human proteases. Proteasix predicts proteases based on the known target sequence specificity, according to the peptidase database MEROPS [41]. The list of predicted proteases was then imported to R and the enrichment over the health dataset was calculated. A hypergeometric test was applied, and the Bonferroni method was used to correct for multiple testing. An adjusted *p*-value < 0.05 was considered significant.

## 3. Results and Discussion

### 3.1. The Urinary Peptidome as a Road to Defining Molecular Disease Signatures

**Figure 1:**
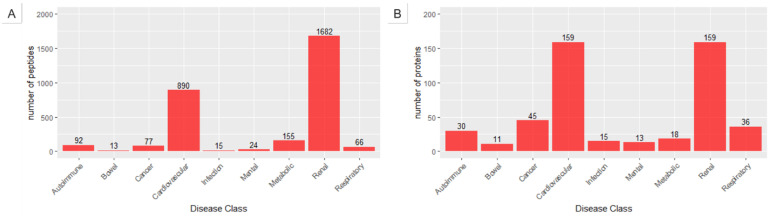
Distribution of unique peptides (**A**) and proteins (**B**) across the different disease classes.

From the human urinary peptidome studies collected and mined, we extracted a total of 3014 differentially expressed peptides in numerous conditions affecting the main physiological systems, such as the renal, cardiovascular, and metabolic systems. Following the volume of studies in those areas, not surprisingly, a higher number of peptides and proteins were found associated with renal (1682 from 159 proteins) and cardiovascular (890 from 159 proteins) diseases (Figure 1). Although we only included one study unequivocally reporting the healthy urine peptidome, the number of sequenced peptides is comparable to the sum of disease peptides: 3765 unique peptides were gathered to represent the healthy urinary peptidome [38]. A quick comparison between disease and health proteome and peptidome shows that while 30% of the proteins (88 out of 294) dysregulated in disease were present in the comprehensive health dataset (Table S4), only 17.4% of the dysregulated of the peptides (203 out of 1166) were also sequenced in the healthy patients. This supports the notion that biomarkers panels composed of peptides can more easily discriminate a disease than a protein panel. Although encouraging, these statistics should be interpreted with caution, since the peptides have been collected from different studies, using diverse experimental approaches (though, not as much regarding sample preparation) and data analysis strategies. Nonetheless, this first analysis: (i) corroborates the vision of urine as an important reservoir of disease biomarkers; (ii) demonstrates the superiority of urine peptidomics to proteomics in biomarker discovery and (iii) suggests that urine’s proteolytic environment underlies the greater diversity of surrogate peptide markers compared to protein markers.

Aiming to uncover and identify peptides with biomarker ability for the 37 conditions studied (spread across nine major classes), a network analysis was performed using Cytoscape. This network can be explored online at NDEx (v.2.5.0) platform https://public.ndexbio.org/#/network/2c846129-a774-11eb-9e72-0ac135e8bacf?accesskey=5ff3875974d153a2b6a8a6bd64988237497a19118713529bb4e9a149ed435960. The conditions are presented as red nodes (source) and the peptide sequences as blue nodes (target). The peptide was mapped according to the degree so that those of first degree (the most specific) were shown as more prominent nodes. As shown by the density of larger blue nodes in the network’s outer layer, there are many peptides with the potential of becoming a signature for many of these diseases–644 in total. This list is provided in Table S6. Beyond being a reservoir of molecular markers for kidney conditions, such as chronic kidney disease (with 43 specific peptides) or type 2 diabetic nephropathy (with 54 specific peptides), this exploratory analysis illustrates that urine is an important source of putative biomarkers for diseases afflicting extra-nephrotic tissues/organs, namely the heart (e.g., heart failure with reduced ejection fraction with 11 specific peptides), the lungs (e.g., chronic obstructive pulmonary disease with 38 specific peptides) or even the bowel (e.g., necrotizing enterocolitis with eight specific peptides).

### 3.2. Can We Distinguish Disease Classes by Peptides’ Physical-Chemical Properties?

**Figure 2:**
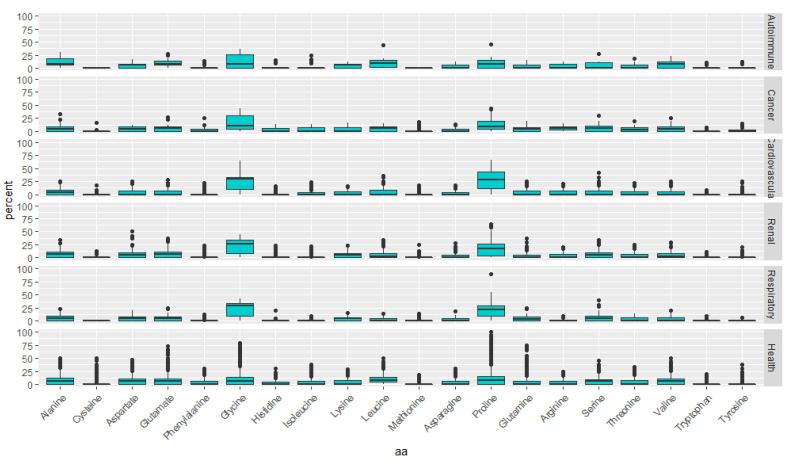
Amino acid composition of each disease class’ “signature” peptides.

Being the most relevant disease-specific urine peptides probed to date, not only regarding health but also considering all other conditions whose the urine peptidome has been investigated, we explored their physical properties in different disease classes hoping to disclose distinctive molecular traits among them. Due to the low representation of some classes in terms of peptides, we only considered the following classes: autoimmune, cancer, cardiovascular, renal, and respiratory diseases. Considering that the amino acids confer the first level of complexity of peptides and are the first determinants of the physical, chemical, and biological properties of peptides, it is not implausible to hypothesize that the amino acid composition, alone, might distinguish different disease classes. Therefore, we started by breaking down the composition of “signature” peptides into the 20 amino acids (Figure 2) to look at potential differences in the primary sequence of peptides assigned to the various classes. Interestingly, autoimmune peptides are characterized by the lack of Cys and Met. Peptides associated with respiratory diseases also lack Cys. One could argue that such an underrepresentation of Cys in autoimmune and respiratory disorders might be an artifact due to the reduction and alkylation of the peptides, but this was not the case. Only in two unrelated studies, one in the setting of renal cell carcinoma [42] and the other in type 1 diabetes mellitus [43] researchers have opted to modify cysteines.

More evident is, perhaps, the higher content in Gly of the disease-associated peptides. A new hypothesis emerges, whereby, the relative increase of Gly on urine peptidome indicts the presence of a disease. Also noticeable is the higher levels of Pro in peptides more specific to cardiovascular and respiratory conditions, suggesting that this amino acid might be a nonspecific marker of these disease classes. Indeed, Alvarez-Llamas et al. [44] have already described the arginine and proline metabolism dysregulation in a leporine model of atherosclerosis after characterizing the urine metabolome.

**Figure 3:**
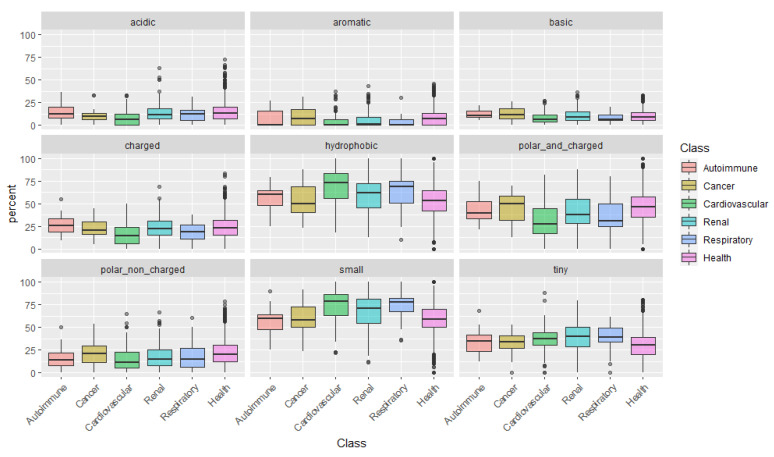
Composition of amino acid groups, according to physical-chemical properties (size, hydrophilicity/hydrophobicity, and isoelectric point) across the five main disease classes.

After examining the amino acids individually, we categorize them according to their physical-chemical properties: acidic, aromatic, basic, charged, hydrophobic, polar and charged, polar non-charged, small, and tiny amino acids (Figure 3). This analysis uncovered a pattern in peptides associated with cardiovascular conditions: peptides are mainly composed of small and more hydrophobic amino acids, with low representation of polar and/or charged amino acids.

**Figure 4:**
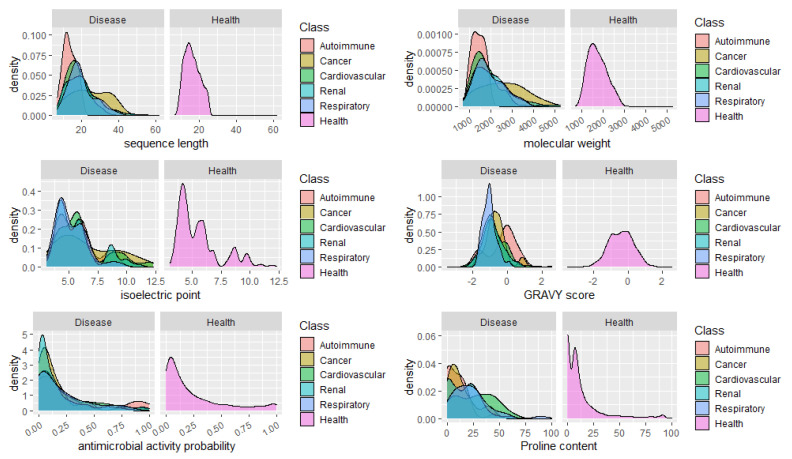
Density plots showing the distribution of peptides in health versus six main disease classes, regarding sequence length, molecular weight, isoelectric point, GRAVY score, antimicrobial peptide probability, and proline content.

Finally, we analyzed the distribution of peptides in health and disease sets concerning relevant features and properties of the peptides, such as the sequence length, molecular weight, isoelectric point, GRAVY score, and the content in Proline. As shown in Figure 4, there are no differences in the distribution of peptides according to their antimicrobial potential, except maybe for a small group of peptides associated to autoimmune disorders, showing a high probability of antimicrobial activity. It would be expected a high percentage of peptides with predicted antimicrobial peptides in urine from patients with infections, however we did not include the peptides associated to infections in this analysis, given the low number of specific peptides found (14). No apparent major changes in the distribution of the peptides regarding the isoelectric point could be seen, as well. Concerning the GRAVY score, a commonly used metric to evaluate a peptide’s hydrophobicity, a very sharp distribution of peptides around -1 (hydrophilic peptides) was observed for respiratory diseases. This might be explained by the fact that hydrophilic peptides are more easily transported in the blood from extrarenal tissues to the kidneys and then more easily filtered off to urine.

Nonetheless, surprisingly, a clear pattern of longer and heavier fingerprint peptides was found for cancer. Could the biological foundation of this observation reside on the operation of more stringent proteases, resulting in lesser cleavage events and, thus, in, averagely longer and heavier peptides? Alternatively (or concomitantly), is this the result of the inhibition of naturally active proteases? Due to the clinical potential, this topic deserves further investigation. Particularly, it would be important to assess if, for instance, in cancer risk models, the addition of the average peptide length or mass on top of clinical variables could improve the model’s predictive power. Finally, the distribution of peptides according to the content in Pro, evidenced once more, the predominance of Pro-rich peptides in cardiovascular diseases. See, for instance, the large proportion of peptides composed of 40–60% Pro in cardiovascular conditions in Figure 4. Whether this feature, complemented with clinical variables, might improve a cardiovascular condition’s diagnosis through urinalysis remains to be confirmed in future studies focused on peptidome and amino acid composition analysis.

### 3.3. Can Proteases Help Distinguish Diseases?

Glancing over the diversity of urinary peptides associated with unrelated pathologies, we hypothesized that predicting the most active proteases would give another layer of discrimination between such conditions. From over 550 proteases composing the human degradome, we predicted the activity of 74 proteases, from four different catalytic types: aspartic (4), cysteine (13), metalloproteases (22), and serine (35) proteases, acting on either the N- or C-termini of the urine proteins/peptides. We started by looking at the proteases which are more susceptible to dysregulation in disease. For such purpose, we computed the fold-enrichment over the proteases predicted to be active in health.

As depicted in Figure S1, we could not calculate the fold-enrichment for two serine proteases from the complete set of predicted proteases as these were only predicted in disease. These were the coagulation factor Xa (F10), responsible for converting prothrombin to thrombin, and the tryptase alpha (TPSAB1), a neutral protease present in mast cells. This analysis also evidences that metalloproteases are the most susceptible to dysregulation, remarkably stromelysin-2 (MMP10), which participates in extracellular matrix disassembly, for example, through fibronectin degradation. Following metalloproteases, serine proteases are the second class more prone to dysregulation, noticeably tripeptidyl-peptidase 1 (TPP1), a lysosomal protease acting mainly on hydrophobic proteins. Cysteine and aspartic proteases complete the list and are the enzymes least susceptible to activation or the proteases leading to minor changes in the urine peptidome in disease. Of note, only three proteases were predicted exclusively from the healthy peptidome: beta-secretase 1 (BACE1), an aspartic protease responsible for the proteolytic processing of amyloid precursor protein, plasma kallikrein (KLKB1), a serine protease with a key role in hemostasis, and trypsin-3 (PRSS3), a serine protease involved in digestion, among other processes. Whether their absence in the disease urine degradome is due to the increased expression of protease inhibitors remains to confirm.

**Figure 5:**
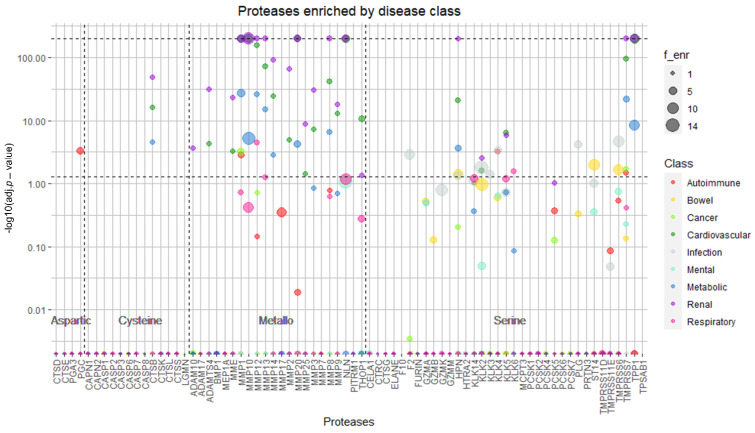
Protease fold-enrichment in the various disease classes: autoimmune (red), bowel (yellow), cancer (light green), cardiovascular (dark green), infection (grey), mental (cyan), metabolic (dark blue), renal (purple), and respiratory (pink) diseases. The size of the circles representing each protease is proportional to the magnitude of the enrichment. Proteases are arranged vertically according to the Bonferroni corrected *p*-value of the enrichment (hypergeometric test). The central horizonal line marks the significance threshold (*p* = 0.05). For representation purposes, proteases showing −log10(*p*-value) > 200 were set to −log10(*p*-value) = 200 and are represented on top of the upper horizonal line.

We hypothesized that different diseases and specific conditions would have distinct degradome profiles by looking at these preliminary data. To investigate this more in-depth, we used a similar strategy to gene ontology enrichment analysis to identify overrepresented proteases compared to health status. Proteases predicted in health were set as the reference library: 61,517 predictions from 3702 peptides, representing 98.2% of the initial peptide dataset. Concerning diseases, a total of 67,159 protease predictions could be made from 3001 peptides, representing 99.6% of all sequences initially identified. To identify which proteases were significantly enriched across diseases/classes, we performed a hypergeometric test and corrected for multiple testing by the Bonferroni method. Figure 5 shows the proteases predicted to be the most active in the various disease classes. We can see some proteases patterns according to the type of disease or the system affected by analyzing this plot. For instance, the aspartic protease gastricsin (PGC) is significantly enriched exclusively in autoimmune diseases. Serine proteases, remarkably hepsin (HPN), suppressor of tumorigenicity 14 (ST14), and transmembrane protease serine 6 (TMPRSS6) are significantly enriched in bowel diseases. Infections are characterized solely by serine proteases’ dysregulation, namely TMPRSS6 and, more specifically, prothrombin (F2) and plasminogen (PLG), well-known hemostasis regulators. In turn, renal, cardiovascular, and metabolic diseases show pronounced dysregulation of metalloproteases, particularly of matrix metalloproteases. The activation of kallikrein-6 (KLK6) is exclusive of respiratory conditions. Perhaps due to cancer’s heterogeneous nature, no clear profile of proteases was observed for this class. Nonetheless, the activation of the interstitial collagenase (MMP1) might be the result of the extracellular matrix disarray, an essential step for tumor invasion and metastasis [45]. Of note, cathepsin B (CTSB), a lysosomal protease involved in intracellular protein catabolism, is the only cysteine protease significantly enriched in disease (class level), but its activation is common to renal, cardiovascular, and metabolic conditions. For mental conditions as a main class, no overall protease enrichment was also evident.

**Figure 6:**
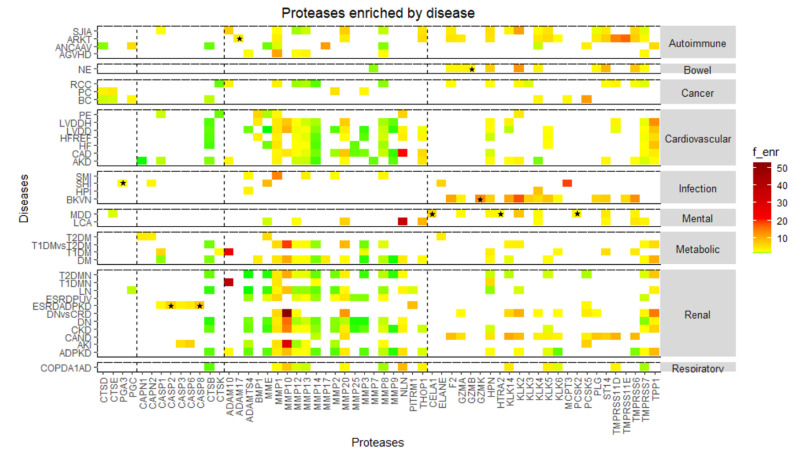
Heatmap showing significantly enriched proteases across specific conditions. Diseases are aligned vertically (please see abbreviations) according to the main class and proteases are aligned horizontally according to the catalytic class. A color code was set for fold-enrichment: 0–1 (under-representation), green; 1–10, yellow; 10–20, orange; 20–30, red; >30 dark red. Stars mark the unique proteases that are enriched exclusively in one disease.

The degradome signature of each disease is depicted in Figure 6. Proteases are sorted horizontally according to the catalytic class. Only proteases showing a significant enrichment are depicted. Each disease is clustered in a panel according to the respective class. This analysis shows that the protease profile increases our insight regarding differences between several conditions, showing exciting clinical potential. For instance, once again, MMP10 stands out as an overactive protease in many conditions, such as renal, metabolic, and cardiovascular diseases. This heatmap also highlights the activation of aspartic proteases in cancer. Also noteworthy is the almost exclusive activation of serine proteases in BK virus nephritis, as well as in necrotizing enterocolitis and in a chronic state of allograft nephropathy/dysfunction. Curiously, an end-stage renal disease in the setting of autosomal dominant polycystic kidney disease is mainly characterized by the activation of caspases.

Aiming at inspecting the specificity of the putative proteases to every disease at scope, we built a map with Cytoscape showing all significant associations as deemed by the hypergeometric test (the reader is referred to https://public.ndexbio.org/#/network/8c07b5f7-a8e3-11eb-9e72-0ac135e8bacf?accesskey=653ffb5202553f1d3db97c4aeac15c0b1500efdf67e70f85a6d7b5478c3901c5) for an interactive exploration of each condition’s putative degradome signature). Proteases (outer circle in the network) are arranged in a counterclockwise ascending spiral, highlighting an increasing specificity. Due to proteases’ participation in many biological processes that are common to many pathological conditions, it is not surprising to verify that only seven out of 37 conditions included in this study can, theoretically, be identified by higher activity of a single protease. For instance, our analysis shows that F10 is explicitly dysregulated in Type 2 diabetic nephropathy. This comes with no surprise, given the association of diabetes with a hypercoagulable state of the blood and the fact that this protease has been pointed as a novel therapeutic target for this condition [46].

Another noteworthy observation is the overactivation of granzyme K (GZMK, 13.6-fold enrichment) in BK virus nephritis. This may be explained by lymphocytes’ activity, which releases granzymes to induce apoptosis of virus-infected cells, often resulting in collateral damage to noninfected tubule cells [47]. In turn, the association of caspase-2 (CASP2, 6.4-fold enrichment) and of caspase-8 (CASP8, 7.8-fold enrichment) activity with end-stage renal disease in the setting of the autosomal dominant polycystic disease has been confirmed in a rat model. It explains the activation of both the intrinsic and extrinsic pathways of apoptosis, a major hallmark of this disease [48]. The protease granzyme B (GZMB, 3.3-fold enrichment), which is also known for its role in stimulating apoptosis, was predicted exclusively from peptides associated with necrotizing enterocolitis, an association that has not yet been reported. According to our analysis, acute rejection of kidney transplant can also be monitored by assessing the activity of the protease disintegrin and metalloproteinase domain-containing protein 17 (ADAM17, 4.1-fold enrichment). ADAM17 has an important pro-inflammatory role in this condition, through shedding of tumor necrosis factor-alpha from its membrane-bound form as well as of its receptors [49]. Moreover, theoretically, a *Schistosoma haematobium* infection may be diagnosed by measuring the activity of urine pepsin A-3 (PGA3, 2.9-fold enrichment). PGA3 may be part of the protease armamentarium of this species that is essential to migrate through the host tissues, a common feature of many parasites [50]. Finally, a major depression disorder is putatively identified by the dysregulation of three proteases: neuroendocrine convertase 2 (PCSK2, 3.4-fold enrichment), the serine protease high temperature requirement protein A2, mitochondrial (HTRA2, 3.0-fold enrichment), and CELA1 (chymotrypsin-like elastase family member 1, 6.5-fold enrichment). PCKS2, for instance is, according to UniProt gene annotation, involved in processing prohormones and neuropeptide precursors, whose dysregulation might lead to depression.

Apart from these seven exceptions, we could sketch a minimal degradome fingerprint for many (21) of the remaining conditions (30), following the same rationale applied to peptides, where often these are integrated into multiplex panels to improve the accuracy of the diagnostic test. In Table 1, one can find the minimal combination of predicted proteases that identify a given disease, signatures which deserve further consideration in future biomarker studies. Even for the eight conditions that failed to show a specific degradome, the combination with specific peptides (Table S6) may be the key to develop a sensitive and specific diagnostic panel. Regardless of the nature of the biomolecules, peptides or proteases herein identified or predicted should, in principle, foster new avenues of research towards biomarker implementation in the era of 3PM [51].

## 4. Study Limitations

Despite of the wealth of peptidome data compiled and reanalyzed in this paper, there are some limitations to acknowledge. First, we cannot appreciate the influence of demographic variables, such as age, sex, or ethnicity, or the effect of pharmacological treatments, in the different disease peptide profiles, as most, if not all, studies collected do not provide single patient peptide sequence profiles, nor their individual characteristics. Second, there are heterogeneities in the study design and in the instrumentation used, reflecting the large span of publication dates (2004–2020). Consequently, the number of peptides associated to any disease in older studies may be lower, biased by the lower resolution and sensitivity of the mass spectrometers available then. To circumvent this limitation, we restricted the physical-chemical analysis of the “signature” peptides to the disease classes showing higher number of associated peptides (>20). Finally, we could only use one study to extract the reference healthy peptidome. In such a study, six urine sample from healthy individuals was analyzed, resulting in more than 4500 sequenced peptides. One should acknowledge, though, that many more urine peptides remain to identify, and that many of these peptides despite not directly associated with disease, can be present in the urine of ill patients. Therefore, the addition of new urine peptidome datasets of healthy individuals is expected to improve the peptidome and degradome profiles herein sketched.

## 5. Conclusions

In the everlasting quest of finding new and more specific disease biomarkers, either in a single or in a multiplex fashion, urine peptidomics and degradomics progressively occupy a relevant position in the most fruitful methodologic approaches. Urine is simultaneously a simpler biological matrix to uncover biomarkers, with less interfering substances than blood-derived products, and a depot of substances from the entire body, virtually allowing to monitor molecular alterations occurring as a consequence of any disease. Furthermore, the peptidome, while technically simpler to analyze, is more complex than the proteome, which may increase the odds for discriminating diseases based on a combination of analytes, not to mention that peptidomics is a good source of information for the characterization of the degradome–itself another source of surrogate markers.

The analysis of all peptidome datasets available today demonstrated its great potential towards biomarker discovery, as shown by the tenths of specific peptides for various conditions afflicting different systems, such as the renal, cardiovascular, pulmonary, and metabolic ones. These peptides are disclosed without reserve as supplementary material and their diagnostic potential merit further scrutiny. Moreover, our analysis suggests that peptides physical-chemical properties may themselves help improve the robustness of disease-predicting models. Particularly, it might be interesting in the future, to test the discriminatory value of proline content for cardiovascular diseases and the value of sequence length/mass for the diagnosis of cancer.

Finally, the present study shows that peptidomics is a double source of information, whose potential extends beyond the insight on dysregulated peptides, by allowing to unveil dysregulated proteases in disease, making use of predictive tools (Proteasix). The prediction of the degradome from the most comprehensive urine peptidome dataset reunited to date revealed a remarkable specificity of the granzymes B and K, caspases 2 and 8, pepsin A-3 and of the disintegrin and metalloproteinase domain-containing protein 17, neuroendocrine convertase 2, serine protease HTRA2 (mitochondrial) and the chymotrypsin-like elastase family member 1 which were associated with a single condition. Furthermore, a specific degradomic signature combining no more than three proteases could be sketched for most of the enrolled conditions, whose validation is imperative. Ultimately, this study advocates the combination of a urine peptidomics-degradomics approach for the discovery and development of new biomarker panels regardless of the origin of the disease. These molecular patterns can potentially be used in any of the three main axes of 3PM, from predicting to preventing and personalizing medical treatment, with the great advantage of a noninvasive monitoring of the disease evolution or remission through a liquid biopsy.

## Figures and Tables

**Table 1:** Putative minimal degradome signature for all conditions studied through urine peptidomics.

Condition ^1^	Class	Minimal Degradome Signature
Type 2 diabetic nephropathy	Renal	F10 ^2^
BK virus nephritis	Infection	GZMK or TPSAB1 ^2^
End-stage renal disease in the setting of autosomal dominant polycystic kidney disease	Renal	CASP2 or CASP8
Necrotizing enterocolitis	Bowel	GZMB
Acute rejection of kidney transplant	Autoimmune	ADAM17
Schistosoma haematobium infection	Infection	PGA3
Major depressive disorder	Mental	PCSK2, HTRA2 or CELA1
Acute Kawasaki disease	Cardiovascular	CAPN1 + MMP7
Bladder cancer	Cancer	CTSE + MCPT3
Lupus nephritis	Renal	PITRM1 + PGC
Renal cell cancer	Cancer	KLK3 + CTSK
Preeclampsia	Cardiovascular	CTSK + BMP1
Diabetes mellitus	Metabolic	MMP17 + BMP1
Autosomal dominant polycystic kidney disease	Renal	KLK6 + MMP9
Left ventricular diastolic dysfunction and hypertension	Cardiovascular	BMP1 + TMPRSS11D
Prostate cancer	Cancer	CTSE + MMP2
Helicobacter pylori infection	Infection	ADAMTS4 + KLK4
Diabetic nephropathy versus chronic renal disease	Renal	GZMA + MMP10
Acute kidney injury	Renal	(CASP3 or CASP6) + (ADAMTS4 or MMP2)
Anti-neutrophil cytoplasmic antibody-associated vasculitis	Autoimmune	MMP17 + CTSD + PGC
Type 2 diabetes mellitus	Metabolic	CAPN1 + CAPN2 + ELANE
Type 1 diabetes mellitus	Metabolic	CTSK + ADAM10 + CASP1
Systemic juvenile idiopathic arthritis	Autoimmune	ADAM10 + CASP1 + F2
Left ventricular diastolic dysfunction	Cardiovascular	MMP9 + TMPRSS11D + THOP1
Chronic kidney disease	Renal	ADAMTS4 + MMP9 + MMP25
Type 1 diabetes mellitus versus Type 2 diabetes mellitus	Metabolic	KLK14 + KLK2 + MMP3
Chronic obstructive pulmonary disease with alpha-1-antitrypsin deficiency	Respiratory	TMPRS11D + KLK6 + NLN
Chronic allograft nephropathy or dysfunction	Renal	GZMA + PCSK5 + (F2 or TMPRS11D)

^1^ Conditions without a unique degradome profile: acute graft-versus-host disease (autoimmune); coronary artery disease, heart failure, heart failure with reduced ejection fraction (cardiovascular); type 1 diabetic nephropathy, diabetic nephropathy, end-stage renal disease in the setting of posterior urethral valves (renal); *Schistosoma mansoni* infection (infection). ^2^ Not shown in the network because it was only predicted from urine peptides identified in pathological conditions.

## Data Availability

All data generated or analyzed during this study is provided as supplementary files. Networks are deposited without reserve in NDex. The reader can explore the peptides and proteases associated with disease by following the links provided in the paper.

## Supplementary Materials

The following are available online at https://www.mdpi.com/article/10.3390/ijms22115940/s1.

## Abbreviations

AGVHD: Acute graft-versus-host disease; AKD: Acute Kawasaki disease; AKI: Acute kidney injury; ARKT: Acute rejection of kidney transplant; ANCAAV: Anti-neutrophil cytoplasmic antibody-associated vasculitis; ADPKD: Autosomal dominant polycystic kidney disease; BKVN: BK virus nephritis; BC: Bladder cancer; CAND: Chronic allograft nephropathy or dysfunction; CKD: Chronic kidney disease; COPDA1AD: Chronic obstructive pulmonary disease with alpha-1-antitrypsin deficiency; CAD: Coronary artery disease; DM: Diabetes mellitus; DN: Diabetic nephropathy; DNvsCRD: Diabetic nephropathy versus chronic renal disease; ESRDADPKD: End-stage renal disease in the setting of autosomal dominant polycystic kidney disease; ESRDPUV: End-stage renal disease in the setting of posterior urethral valves; HF: Heart Failure; HFREF: Heart failure with reduced ejection fraction; HPI: *Helicobacter pylori* infection; LCA: Low cognitive ability; LVDD: Left ventricular diastolic dysfunction; LVDDH: Left ventricular diastolic dysfunction and hypertension; LN: Lupus nephritis; MDD: Major depressive disorder; NE: Necrotising enterocolitis; PE: Preeclampsia; PC: Prostate cancer; RCC: Renal cell cancer; SHI: *Schistosoma haematobium* infection; SMI: *Schistosoma mansoni* infection; SJIA: Systemic juvenile idiopathic arthritis; T1DM: Type 1 diabetes mellitus; T1DMvsT2DM: Type 1 diabetes mellitus versus type 2 diabetes mellitus; T1DMN: Type 1 diabetic nephropathy; T2DM: Type 2 diabetes mellitus; T2DMN: Type 2 diabetic nephropathy.
