# Supplementary figures and images for: Mining the Biomarker Potential of the Urine Peptidome: From Amino Acids Properties to Proteases
Source: Int J Mol Sci. 2021 May 31;22(11):5940. doi: 10.3390/ijms22115940 (PMC8197949; doi:10.3390/ijms22115940)

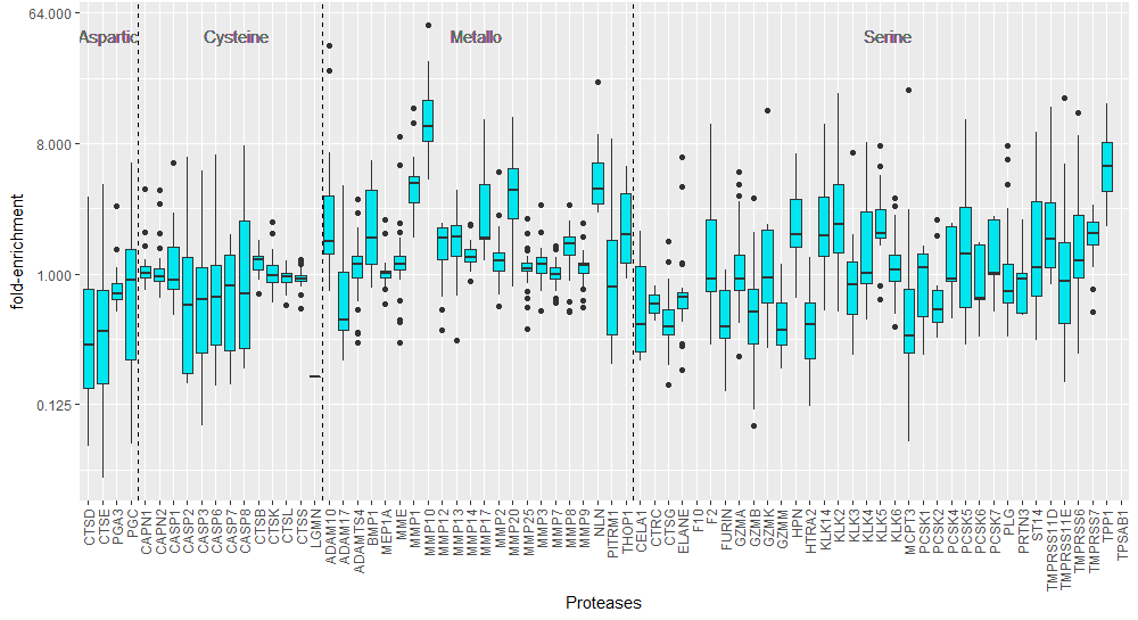

Supplement: Supplementary file 1 [file ijms-22-05940-s001.zip › Supplemental Figure 1.tif]
